# Beneficial Effects and Safety of Corticosteroids Combined with Traditional Chinese Medicine for Pemphigus: A Systematic Review

**DOI:** 10.1155/2015/815358

**Published:** 2015-02-22

**Authors:** Tingting Zhou, Peiru Zhou, Hong Hua, Xiaosong Liu

**Affiliations:** Department of Oral Medicine, Peking University School and Hospital of Stomatology, Beijing 100081, China

## Abstract

*Objective*. To evaluate the beneficial effects and safety of corticosteroids combined with traditional Chinese medicine (TCM) for pemphigus. *Methods*. Seven electronic databases were searched to identify any potential randomized controlled trials (RCTs) or clinical controlled trials (CCTs) that compared corticosteroids with and without TCM for the treatment of pemphigus, published in any language. Remission of the mucocutaneous lesions, therapeutic duration, dosage of corticosteroids, and specific antibody titers were employed as the main outcome measures. The methodological quality of the included studies was assessed using the Cochrane Handbook for Systematic Review of Interventions and Rev Man 5.1.0 software. *Results*. Four RCTs with a total of 199 patients were included in the present review. Management with corticosteroids combined with TCM seemed to benefit pemphigus patients in terms of healing of lesions, prevention of complications and relapse, and reduced interferon-gamma (IFN-*γ*) level. The trials were not of high methodological quality. No study mentioned allocation concealment and blinding. Only one trial reported adverse events, and it indicated that the safety of corticosteroids combined with TCM was uncertain. *Conclusion*. There is some, albeit weak, evidence to show that combined treatment with corticosteroids with TCM could be of benefit for some patients with pemphigus. The efficacy and safety of this combined treatment should be evaluated further in better designed, fully powered, and confirmatory RCTs.

## 1. Introduction

Pemphigus is a rare group of autoimmune bullous diseases characterized by widespread blistering and erosions on the skin and mucous membranes. The incidence of pemphigus has been estimated to be 1 to 16 new cases per million people per year [1]. Antibodies against desmoglein-1 and/or desmoglein-3 are the main cause of these diseases [2].

Pemphigus is a chronic and potentially life-threatening condition [3]. With the introduction of corticosteroids in the 1950s, the mortality rate of pemphigus patients decreased from 75% to 30% [4]. To date, systemic treatment with corticosteroids is recommended clinically as the first-line remedy. However, high doses of and prolonged exposure to systemic corticosteroids, together with the use of adjuvant immunosuppressive and anti-inflammatory agents, cause severe adverse effects in PV patients [5], such as Cushing syndrome, necrosis of the femoral head, and digestive bleeding. To minimize the side effects of corticosteroids, traditional Chinese medicine (TCM) has been used as an adjuvant in China since the 1980s [6]. Many clinical studies have shown that it can effectively improve patients' conditions and therapeutic efficacy and reduce corticosteroid dosage, complications, and the risk of recurrence [7–10]. However, no evidence-based studies using corticosteroids combined with TCM for treatment of pemphigus have appeared in the literature. In this analysis, randomized controlled trials (RCTs) and clinical controlled trials (CCTs) were collected to evaluate the beneficial effects and safety of corticosteroids combined with TCM for pemphigus. 

## 2. Materials and Methods

### 2.1. Database and Search Strategies

We selected all clinical trials that compared corticosteroid treatment with combined treatment of corticosteroids and TCM for treatment of pemphigus in the Chinese National Knowledge Infrastructure (CNKI), the Chinese Biomedical Literature Database (CBM), the Chinese Scientific Journal Database (VIP), WANFANG, PubMed, EMBASE, SCI, Current Controlled Trials, and the Cochrane Central Register of Controlled Trials in the Cochrane Library (March 2014). No restrictions were placed on language. The following search terms were used individually or combined: “Chinese patent medicine,” “Chinese patent drugs,” “traditional Chinese medicine,” “Chinese herbology,” “Chinese medicine,” “Chinese material medical,” “Chinese herbs,” “Chinese herbal medicine,” “herbal medicine,” “Chin Tradit Pat Med,” corticoid, corticosteroids, “rat ATH,” glucocorticoid, “cortical hormone,” pemphigus, “controlled clinical trial,” “clinical trial,” and “randomized controlled trials.”

### 2.2. Inclusion and Exclusion Criteria and Process

*Inclusion Criteria.* RCTs and CCTs that compared corticosteroids with and without TCM for pemphigus were collected. Based on the “Diagnosis and classification of pemphigus and bullous pemphigoid” [11], patients fulfilling the diagnostic criteria for pemphigus were enrolled, without restrictions on age, gender, or race. Outcome measures included clinical outcome (e.g., remission of the lesions, dosage of the corticosteroid, and complications) and laboratory outcome (e.g., titers of Dsg1 and 3 and IIF).

*Exclusion Criteria.* Exclusion criteria were as follows: duplicate publications reporting the same groups of participants; case reports, reviews, workshop summaries, and studies about clinical observations; studies that included pemphigoid or other autoimmune bullous diseases; research reports without relevant information on patients and interventions; studies that included any adjuvant (i.e., azathioprine, methotrexate, or steroid sparing agents) before starting TCM.

### 2.3. Data Extraction and Quality Assessment

Two authors (Tingting Zhou and Peiru Zhou) were responsible for searching the literature, selecting studies, and extracting data independently. Various data were extracted including the title of the study, author, year of publication, article source, study size, sample size, diagnostic criteria, methodological details, therapeutic duration, and clinical standards, as well as interventional details of controls, outcomes, and adverse effects for each study. Disagreements were resolved by discussion, and consensus was achieved through a third party (Hong Hua, Xiaosong Liu). The quality of the enrolled trials was assessed according to the Cochrane Handbook for Systematic Review of Interventions, Version 5.1.0, and Rev Man 5.1.0 software [12]. The assessment criteria used were random sequence generation (selection bias), allocation concealment (selection bias), blinding of participants and personnel (performance bias), blinding of outcome assessment (detection bias), incomplete outcome data (attrition bias), selective reporting (reporting bias), and other bias.

### 2.4. Data Synthesis

A descriptive analysis of the data was conducted in this systematic review because of statistical heterogeneity and the limited number of patients enrolled.

## 3. Results

### 3.1. Description of the Included Studies

**Figure 1:**
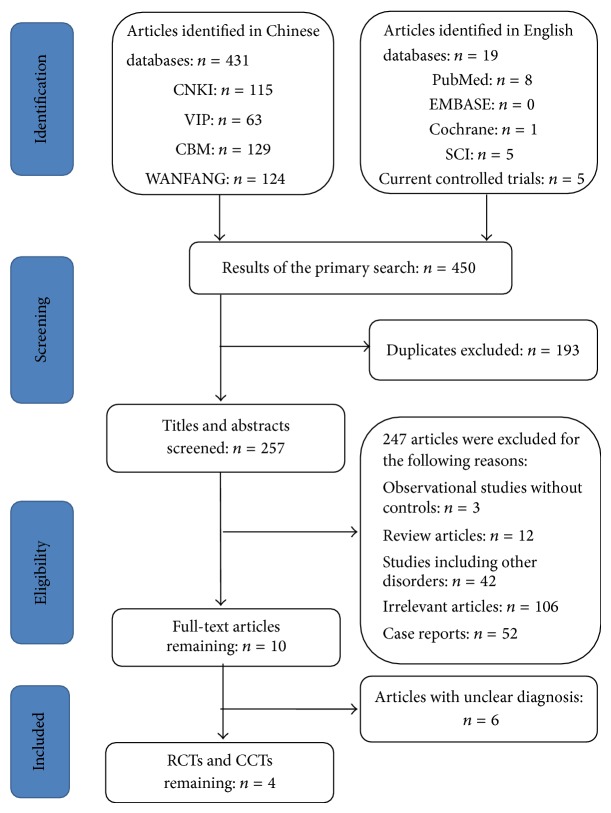
Study selection process.

A total of 450 abstracts (431 in Chinese and 19 in English) were obtained from 9 databases and were evaluated on the basis of the inclusion criteria of the present analysis.  Figure 1 shows details regarding the full flow of information, presented according to the PRISMA format [13]. A total of 193 studies were excluded for duplication. Among the 257 remaining abstracts, 106 abstracts were not relevant, and 42 described bullous disorders other than pemphigus. Therefore, 10 full-text articles were reviewed. Further examination showed that only four trials met the inclusion criteria; they were included in the present analysis [6, 14–16].

### 3.2. Characteristics of Studies

Table 1 shows the characteristics of the four clinical trials included in this review. A total of 199 pemphigus patients were studied in these trials. Of these patients, 103 were prescribed a combination of oral corticosteroids and TCM, while 96 patients were treated with corticosteroids only. The sample size in each trial ranged from 20 to 32.

### 3.3. Risk of Bias and Quality Assessment of Studies

**Figure 2:**
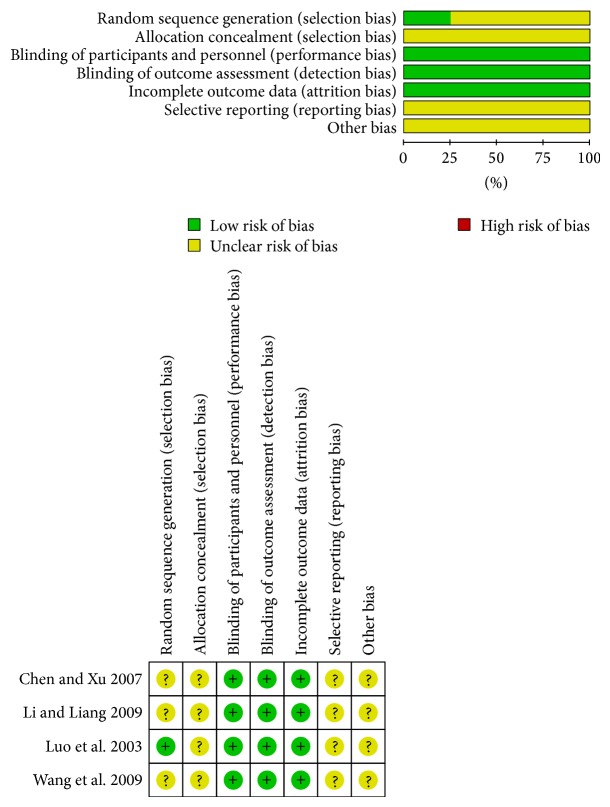
The Cochrane Collaboration's tool for assessing risk of bias.

The quality of most trials was poor according to the Cochrane quality assessment criteria (as shown in Table 2).  Figure 2 shows the risk of bias. No information about allocation concealment, blindness, or withdrawals and dropouts was recorded in any trial. Only one trial reported the randomized allocation of participants, follow-up, and adverse reactions.

### 3.4. Effects of the Interventions

To summarize the data, a descriptive analysis was conducted due to the limitations of both statistical heterogeneity and the number of articles analyzed.

### 3.5. Main Outcome Measure

The remission of mucocutaneous lesions, therapeutic duration, dosage of corticosteroids, complications, and relapse were evaluated as main outcome measures.

#### 3.5.1. Remission of Lesions

Three out of four trials reported the remission of lesions. In Li's study, the improvement of blisters was significant (*t*-test, *P* < 0.05) in the group with TCM, while no significant difference was demonstrated between groups in fever or causalgia [14]. The response of the pemphigus patients in the active condition was documented by Peng and Jie [15]. After 2 months of treatment, only 6.25% (2/32) and 24.14% (7/29) of the patients remained unchanged in the groups with and without TCM, respectively. The ratio of improvement in the group with TCM was significantly higher than that in the group without TCM (*P* < 0.05). In the third trial [13], 4.5% (1/22) and 35% (7/20) of the patients relapsed during the follow-up period (2 years) in the group with and without TCM respectively (*χ* ^2^ test, *P* < 0.05).

#### 3.5.2. Time of Treatment

Two trials described the therapeutic duration. The size of lesions was measured at different time points of treatment in one trial [14]. In this trial, therapeutic effects were classified into three grades: cured: all previous rashes faded without any new eruption; improved: more than 30% of rashes faded and new rashes appeared occasionally; and not improved: less than 30% of rashes faded and new rashes appeared all the time [17, 18]. Significant differences in the percentage of cured patients at various time points of treatment (2 months, 4 months, and 6 months) were observed between groups (*t*-test, *P* < 0.01). The other trial [6] just mentioned that there was a significant difference between groups in healing time of the lesions (*P* < 0.05), but no detailed times were presented.

#### 3.5.3. Dosage of Corticosteroids

Dosage of corticosteroids was reported in only two trials. In Chen's study [15], no significant difference between the groups was found in the percentage of patients taking less than 30 mg of prednisone per day at the end of 2 months' treatment, although the ratio was higher in the TCM group than in the non-TCM group. However, the difference was significant (*P* < 0.01) when the dosage was more than 30 mg per day. The other trial [6] only mentioned that there was a significant difference (*P* < 0.05) in corticosteroid dosage when the lesions could be controlled well, but no exact figure was given.

#### 3.5.4. Complications

Two trials reported complications. In one study, during treatment, gastric ulcer occurred in two patients, and fungal infection and osteoporosis developed in one and three patients, respectively, in the TCM group. In the group without TCM, 5 patients suffered from gastric ulcer, 7 patients suffered from fungal infection, and 10 patients suffered from osteoporosis. A *t*-test demonstrated a highly significant difference between groups (*P* < 0.01) [14]. In the other study, osteoporosis and hypertension were observed in one patient receiving TCM, while in the group receiving corticosteroids alone, two patients with osteoporosis, three patients with hypertension, two patients with diabetes mellitus, and one patient with digestive tract ulcer were observed (chi-square test, *P* < 0.05). The chi-square test demonstrated a significant difference between the two groups (*P* < 0.05) [16].

#### 3.5.5. Relapse Rate

Only one trial reported the relapse rate [14]. Two patients receiving TCM and six patients receiving corticosteroid alone experienced relapse. A *t*-test demonstrated a highly significant difference between the two groups (*P* < 0.01).

### 3.6. Secondary Outcome Measure

Antibody titer in circulation was set as a secondary outcome measure. One [6] of the four studies compared the titer of specific antibodies for pemphigus between groups, as well as the levels of interleukin-10 (IL-10), interferon-gamma (IFN-*γ*), and soluble interleukin-2 receptor (slL-2R). Prior to treatment, no differences were detected between groups in circulating levels of these markers. However, the levels of IFN-*γ* decreased remarkably in both groups after treatment. The reduction in the group with TCM was much more remarkable than that in the group without TCM (*P* < 0.05). In addition, the level of IL-10 increased in the combined TCM group more than that in the corticosteroid group, but there were no significant differences (*P* > 0.05).

### 3.7. Adverse Events

One case of diarrhea was observed in the combined group by Luo et al. [6]. No other adverse events were reported.

### 3.8. Subgroup Analysis and Publication Bias

The number of trials in the present review was too small to conduct analyses of subgroups and publication bias.

## 4. Discussion

Pemphigus is an acquired autoimmune blistering disease, in which the immune system becomes dysregulated and produces antibodies against normal mucocutaneous components. The use of systemic corticosteroids has dramatically reduced mortality from this disease, but treatment outcome is still associated with profound corticosteroid-related morbidities. To minimize the side effects of these medicines, TCM has been used in China since the 1980s. Yu et al. [7] reported that a combination of corticosteroids and TCM can effectively improve patients' conditions and therapeutic efficacy and reduce corticosteroid dosage and complications. Wu et al. [10] found that this therapeutic strategy could reduce the toxicity of corticosteroids and promote drug absorption. Based on traditional Chinese medicine, pemphigus is attributed to damp, heat, and toxin, which result in the holistic Yin-yang imbalance and dysfunction in the visceral organs of the patient and consequently cause various clinical manifestations [19]. Unlike Western medicine, the core idea of traditional Chinese herbal medicines is to correct the holistic condition, such as with* Coptidis Rhizoma*,* Radix Scutellariae*, and* Cortex Phellodendri*, which are most commonly used for heat-clearing and detoxicating. Modern clinical trials have demonstrated that some herbal medicines have immunosuppressive and anti-inflammatory properties [20].

In the present analysis, four clinical trials consisting of 199 patients were evaluated. Compared to corticosteroids alone, management with corticosteroids combined with TCM seemed to benefit pemphigus patients in terms of healing of lesions, prevention of complications and relapse, and reducing levels of IFN-*γ*. However, conclusions could not be drawn because of several limitations in the present analysis. First, only a few clinical trials were available, and they were small in size and poor in quality of study design. Second, publication bias resulting from the difficulty in publishing negative results may have occurred. Third, a meta-analysis could not be performed because of the small number of available studies and statistical heterogeneity. Nevertheless, the alternative strategy of using corticosteroids combined with TCM for pemphigus may be considered one option to prevent complications, especially when patients are experiencing side effects from corticosteroid treatment.

However, to date, no well-designed or high-quality RCT on the safety and efficacy of combining steroid and TCM treatment for pemphigus has been reported. This analysis suggests that high-quality RCTs are essential to demonstrate the beneficial effects of Chinese herbs for patients with pemphigus. To achieve a strong clinical trial, clinical practitioners should follow the international standards of CONSORT in assessing TCM [21]. Furthermore, this analysis suggests that high-quality RCTs are essential to TCM clinical investigation. In order to achieve an ideal clinical trial, clinical practitioners should follow the international standards of CONSORT for TCM [19]. To make sure that the rationale of the study design is the first step. The study design model, such as the TCM syndrome-oriental model and the integrated syndrome and disease-oriental model, could have effect on the inclusion and exclusion criteria, the treatment protocols, and the outcomes. Secondly, the information of the TCM which included the TCM formula, dosage, treatment course, control intervention, outcome measurement method, and index should be defined clearly. Thirdly, the primary and secondary outcomes and selecting the rationale ones which could improve the reliability of the assessment also should be defined clearly. In addition, statisticians should participate throughout the research process and be responsible for calculating sample size, monitoring the performance of the research, and analyzing data.

## 5. Conclusion

There is some evidence on the use of corticosteroids combined with TCM in promoting healing of lesions, reducing circulating specific antibody levels, and decreasing adverse events and relapse. However, considering the quality of experimental designs and methodologies in existing studies, the evidence remains weak. More rigorous RCTs with high-quality study designs are needed to assess whether corticosteroids combined with TCM are an effective and safe treatment of pemphigus.

## Figures and Tables

**(a):** 

Study	Authors	Year	Article source	Type of study	Sample size (treatment/control)	Diagnosis standard
[14]	Li and Liang	2009	CJCM2009 Vol. (1)	RCT	29/27	Clinical manifestation histopathology and DIF
[15]	Chen and Xu	2007	Journal of New Chinese Medicine, January 2007 VoI. 39 No.1	RCT	32/29	Clinical manifestation histopathology and DIF
[6]	Luo et al.	2003	Journal of Clinical Dermatology, 2003; 32(1): 38–41.	RCT	20/20	Clinical manifestation histopathology and DIF
[16]	Wang et al.	2009	Journal of China Traditional Chinese Medicine Information, April 2009, Vo1. 1 No. 2	RCT	22/20	Clinical manifestation histopathology and immunopathology

**(b):** 

Study	Intervention	
	Treatment	Control
[14]	*Systemic therapy*: (Radix rehmanniae, cornu bubali, coptidis rhizoma, *Gardenia*, radix scutellariae, red peony root, phillyrin, cortex moutan, folium phyllostachytis, herba artemisiae scopariae, *Fritillaria cirrhosa*, caulis akebiae, acorus tatarinowii, rhizoma belamcandae, mechanism, agastache rugosus, cardamom) combined 20 mg dexamethasone iv, regularly reduced after seven days, 5 mg for maintenance and stopped when lesion was controlled.* Topical therapy:* (gallnut, fructus mume, pepper, chaulmoogra, cochinchina momordicae seed, calamine, *Hibiscus syriacus*, *Sophora flavescens*, cortex dictamni, senecio scandens) decoction for external application 3-4 times for one day.	*Systemic therapy*: 12 mg prednisone, once a day, appropriate antibiotics and protecting stomach medicine added according to the symptoms. *Topical therapy*: Gentian violet, nitrofurazone solution, permanganate armour, rivanol gauze for external application.

[15]	(White atractylodes rhizome, raw Gordon euryale seed, cortex phellodendri, *Paeonia suffruticosa*, raw hovenia dulcis, raw semen coicis, SevenlobedYam Rhizome, herba artemisiae scopariae, honeysuckle, cortex sclerotii poriae) combined prednisone.	Oral administration of prednisone.

[6]	Pemphigus I (Copitidis rihizoma, radix scutellariae, cortex phellodendri, *Gardenia*, honeysuckle, glabrous greenbrier rhizome, radix rehmanniae, coix seed, rhizoma atractylodis) combined glucocorticoids.	Glucocorticoids only

[16]	Tonifying kidney herb (Liuweidihuang pill) combined small doses of corticosteroids for maintenance treatment.	Only small doses of corticosteroids for maintenance treatment

**(c):** 

Study	Treatment course	Outcome measure		Complications	Follow-up	Adverse event
		Clinical standards	Laboratory standards			
[14]	More than six months	① The regression of the blister, fever, and the causalgiar, ② the treatment time, ③ relapse	No	Gastric ulcer, fungal infection, and osteoporosis	Not mentioned	Not mentioned

[15]	Two months	① The regression of the blister, ② the dosage of corticosteroids	No	No	Not mentioned	Not mentioned

[6]	Not mentioned	(1) The time used when the lesion was controlled, (2) the highest dosage of corticosteroids used when the lesion was controlled	The titer of the antibodies and the level of IL-10, IFN-*γ*, and slL-2R	No	Not mentioned	One patient with subtotal gastrectomy experienced diarrhea.

[16]	14 days as one course of treatment, just one course every month	The lesion control rate (patients in stable condition and regressed)	No	Osteoporosis, hypertension, diabetes, and peptic ulcer	Two years	Not mentioned

**Table 2:** Quality assessment of included randomized controlled trials.

Study	Random sequence generation	Allocation concealment	Blinding of participants and personnel	Blinding of outcome assessment	Incomplete outcome data	Selective reporting	Other bias	Summary
[14]	Uncertain	Uncertain	Low bias	Low bias	Low bias	Uncertain	Uncertain	Unclear risk of bias
[15]	Uncertain	Uncertain	Low bias	Low bias	Low bias	Uncertain	Uncertain	Unclear risk of bias
[6]	Low bias	Uncertain	Low bias	Low bias	Low bias	Uncertain	Uncertain	Unclear risk of bias
[16]	Uncertain	Uncertain	Low bias	Low bias	Low bias	Uncertain	Uncertain	Unclear risk of bias
